# Endoscopic‐Assisted Division of Septal Formation After Duhamel Procedure for Hirschsprung Disease: Two Case Reports

**DOI:** 10.1111/ases.70334

**Published:** 2026-07-02

**Authors:** Shohei Takami, Shunji Nonaka, Hideyuki Yokokawa, Ayumi Kitahara, Shinzo Yamamoto, Kazuko Obana, Yoshiakira Kanai, Jun Fujishiro

**Affiliations:** ^1^ Department of Pediatric Surgery Japanese Red Cross Medical Center Tokyo Japan; ^2^ Department of Pediatric Surgery The University of Tokyo Hospital Tokyo Japan; ^3^ Department of Veterinary Anatomy The University of Tokyo Tokyo Japan; ^4^ Department of Gastroenterology Japanese Red Cross Medical Center Tokyo Japan

**Keywords:** Duhamel procedure, endoscopy, Hirschsprung disease, septal formation

## Abstract

The management of septal formation after the Duhamel procedure in Hirschsprung disease remains unclear. We report two cases treated with an endoscopic‐assisted approach. Case 1 is a 5‐year‐old girl with Hirschsprung disease who underwent the Duhamel procedure. Persistent constipation led to a contrast enema, confirming septal formation. Transanal endoscopic‐assisted division with an endoscopic linear stapler improved symptoms initially. However, constipation and enterocolitis with bleeding recurred 5 months later, requiring redo surgery. Case 2 is a 21‐year‐old female with a history of Hirschsprung disease, treated with the Duhamel procedure. She had three episodes of severe abdominal pain and enterocolitis in the past month. Colonoscopy revealed a septal formation with proximal intestinal dilatation. Endoscopic‐assisted division resulted in complete resolution of symptoms. No complications occurred. Septal formation may cause an obstruction contributing to constipation and enterocolitis. Early recognition and division of the septum are recommended in patients with severe constipation or enterocolitis after the Duhamel procedure.

## 1 Introduction

Hirschsprung disease (HD) is a congenital disorder presenting with ileus or constipation during infancy. The pathophysiology of HD involves the absence of ganglion cells in a continuous distal intestinal segment extending proximally from the anus, causing impaired bowel motility. Definitive management involves surgical division of the aganglionic segment. Three major surgical procedures are widely employed: the Soave, the Swenson, and the Duhamel [1]. The Duhamel procedure minimizes urethral and pelvic nerve injury by preserving part of the ventral aganglionic rectal wall near the anus and creating a side‐to‐side anastomosis with the normal bowel. Even though no significant differences in surgical outcomes have been reported among the three procedures, the Duhamel technique is associated with a unique late complication: septal formation at the anastomotic site [2]. Septal formation may contribute to postoperative constipation and enterocolitis, but reports on its management are limited.

In this report, we present two cases of septal formation following the Duhamel procedure that were divided using colonoscopic assistance and discuss clinical factors affecting outcomes, including chronic dilation.

## 2 Case Presentation

### 2.1 Case 1

**FIGURE 1:**
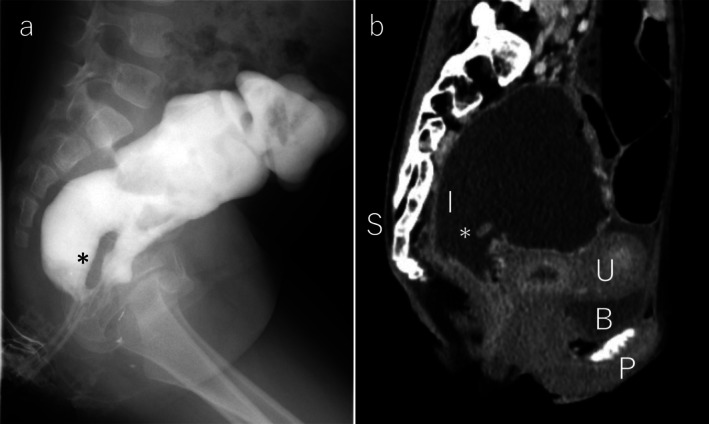
Septal formation after the Duhamel procedure (a) Contrast enema of Case 1. (b) Sagittal enhanced computed tomography of Case 2. *: Septal formation, B, bladder; P, pubis; R, rectum; S, sacrum; U, uterus.

**FIGURE 2:**
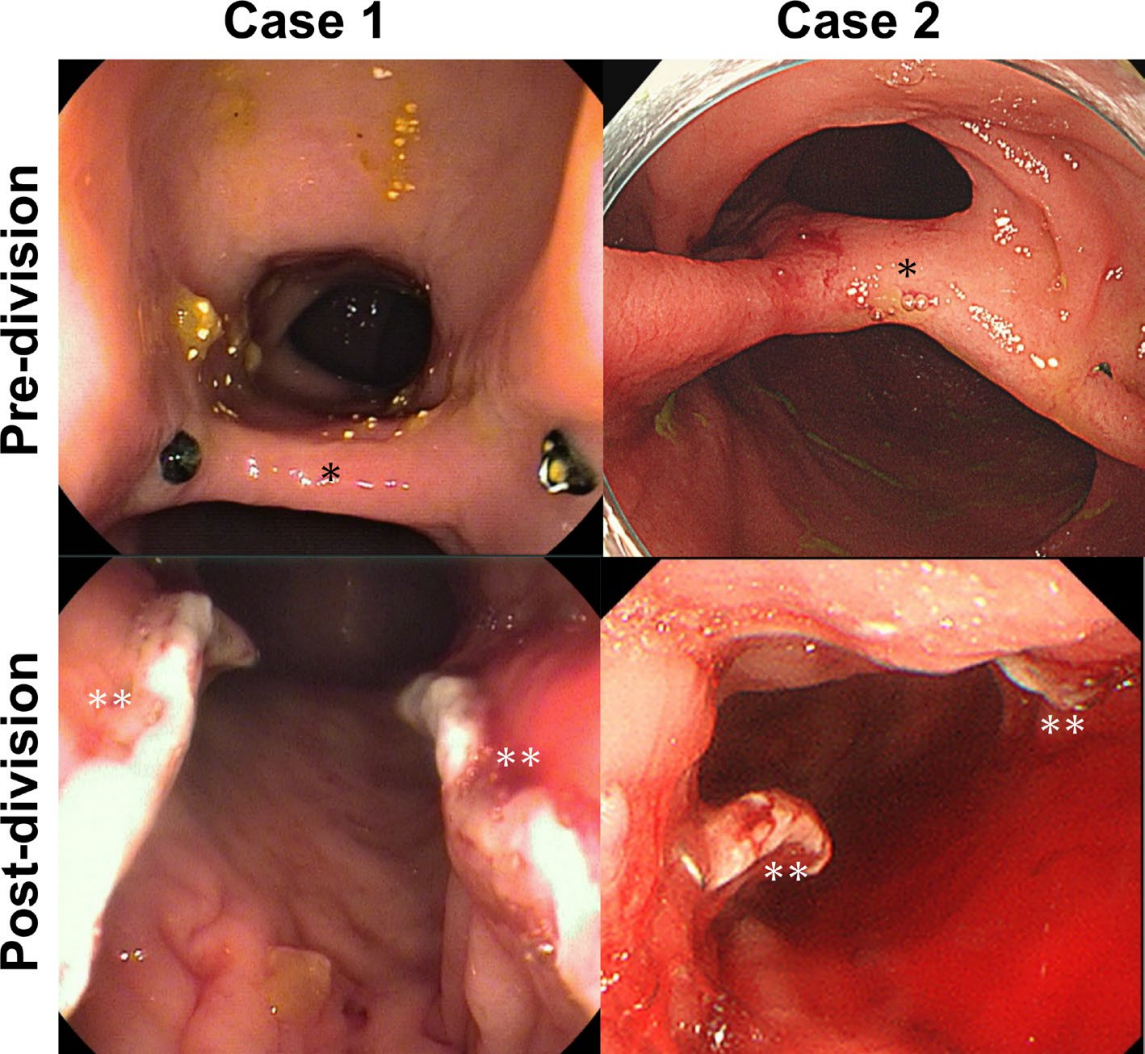
Endoscopic‐assisted division of the septal formation. Upper panels: Case 1. Lower panels: Case 2. Left panels: Pre‐division findings. Right panels: Post‐division findings. *: Septal formation; **: Staple lines.

A 5‐year‐old girl presented with a history of short‐segment HD, situs inversus, and mitral atresia. She underwent a Fontan procedure at age 3 for her congenital heart disease and has been on aspirin therapy since. She underwent the Duhamel procedure using a linear stapler at 6 months of age. However, she developed severe and persistent constipation around age 3. Despite ongoing medical management, the constipation remained refractory to treatment. Contrast enema revealed a septal formation at the anastomotic site, likely contributing to worsening constipation (Figure 1a). Consequently, endoscopy‐assisted septotomy using an endoscopic linear stapler was performed. Briefly, a thin flexible endoscope (Olympus GIF‐1200N) was inserted transanally. A Penrose drain was then inserted from the anus and passed dorsal–proximal–ventral to the septal formation so that both ends exited through the anus. Outside the body, the Penrose drain was placed over the cartridge of an endoscopic linear stapler (Ethicon Powered Echelon Flex, Gold cartridge, 45 mm), which was subsequently inserted transanally. Under endoscopic visualization, the septal formation was grasped and divided (Figure 2, upper row). To prevent air leakage from the anus during the procedure, the anal verge was gently sealed with wet gauze. The septum measured approximately 2 cm in length and 5 mm in thickness. The staple line was left intact without additional reinforcement. Oral intake was initiated on the day of surgery, and no intraoperative or immediate postoperative complications occurred. Although constipation improved temporarily after the procedure, it subsequently worsened. Additionally, she experienced recurrent bleeding from the staple line, necessitating the interruption of aspirin therapy. Due to these complications, resection of the dilated intestine and aganglionic rectum and a redo pull‐through procedure using a Soave‐equivalent approach was performed 1 month after septal division.

### 2.2 Case 2

A 21‐year‐old female patient underwent the Duhamel procedure for extended HD at age 1. She had been free from enterocolitis for over 10 years; however, at 21, she had three severe episodes requiring hospitalization, intravenous antibiotics, and transanal fecal drainage. Colonoscopy and enhanced computed tomography revealed septal formation at the anastomotic site with proximal bowel dilation (Figures 1b and 2a). The septum measured approximately 2 cm in length and 5 mm in thickness. Endoscopic‐guided septotomy using the same technique as in Case 1 was performed to divide the septum (Figure 2, lower row). The postoperative course was uneventful, and the patient has since remained free of recurrent enterocolitis.

## 3 Discussion

Septal formation is a late complication of the Duhamel procedure. Several studies have reported incidences ranging from 2.8% to 23.5% [3, 4]. Treatment options include direct resection, linear stapler division, or energy‐device division [5, 6]. To the best of our knowledge, only one study has reported the transanal endoscopy‐assisted division of the septum using an endoscopic linear stapler, similar to the approach described in this report [7]. However, no previous reports have detailed the technical aspects and outcomes of this approach.

A technical advantage of this approach is the ability to visualize the septum directly using a flexible endoscope while guiding the stapler transanally. The Penrose drain facilitated safe traction and positioning of the septum into the stapler cartridge. In addition, endoscopic confirmation enabled avoidance of injury to the opposite bowel wall.

Compared with direct resection via laparotomy, this approach is less invasive and avoids opening the bowel lumen, thereby reducing the risk of intra‐abdominal contamination. Transanal division using an energy device is similarly minimally invasive; however, there is a potential risk of unintended thermal injury to adjacent tissues due to manipulation within a narrow intestinal lumen. Although the present approach is minimally invasive and carries a lower risk of injury to surrounding structures, Case 1 demonstrated an unfavorable course with recurrent bleeding. The staple line may have contributed to recurrent bleeding in Case 1. Stapler‐associated bleeding has been reported following the Duhamel procedure as well as in other stapling techniques [8, 9]. Staples may induce local ischemia, foreign body reactions, and mechanical irritation, all of which can contribute to ulcer formation and subsequent bleeding [9]. Therefore, the use of staplers should be carefully considered in patients with a high risk of bleeding, such as those receiving antiplatelet therapy, as in Case 1. Although not previously reported in this context, direct septal resection with mucosal suturing using transanal minimally invasive surgery (TAMIS) may represent a feasible alternative in selected high‐risk cases [10].

In contrast, Case 2 showed complete resolution of symptoms without complications, suggesting that this approach may be particularly effective in patients without comorbidities or bleeding tendencies.

We also developed a simplified fluid dynamics model of septal division (Supporting Information). This simplified model may suggest that septal division is more effective in cases with minimal intestinal dilation and softer stool consistency. In Case 2, the relatively short duration of symptoms and the reversibility of bowel dilation, along with the presence of extended Hirschsprung disease and relatively loose stool consistency, may have contributed to the favorable outcome.

## 4 Conclusion

Endoscopy‐assisted septal division is a minimally invasive therapeutic option for managing septal formation after the Duhamel procedure. However, given the potential for stapler‐related complications and suboptimal outcomes in certain cases, this technique should be selectively applied after careful consideration of alternative treatment options.

## Author Contributions

S.T., H.Y., A.K., S.Y., and K.O. were involved in the clinical management of the patient. S.T. and H.Y. collected the clinical data. S.T. performed the literature review and wrote the first draft of the manuscript. S.N. reviewed the validity of fluid dynamics model. Y.K. and J.F. critically reviewed and revised the manuscript. All authors read and approved the final version of the manuscript.

## Funding

This study was supported by the Young Investigator's Award from the Japan Surgical Society (S.T.).

## Ethics Statement

This report was conducted in accordance with the ethical guidelines in Japan, which state that institutional review board approval is not necessary for case reports. Written informed consent was obtained from the patients (or their legal guardians) for publication of this report and accompanying images.

## Conflicts of Interest

The authors declare no conflicts of interest.

## Supporting information


**Figure S1:** Fluid dynamics model of intestinal stenosis with the septal formation *L*: length, *D*_anat_: the diameter of the anastmotic site with the septum, Din: the original intestinal diameter.

**Data S1:** Supporting Information.

## Data Availability

The data that support the findings of this study are available on request from the corresponding author. The data are not publicly available due to privacy or ethical restrictions.
