# Supplementary material for: Endoscopic‐Assisted Division of Septal Formation After Duhamel Procedure for Hirschsprung Disease: Two Case Reports
Source: Asian J Endosc Surg. 2026 Jul 2;19(1):e70334. doi: 10.1111/ases.70334 (PMC13328324; doi:10.1111/ases.70334)
Supplement: Supplementary file 1 — Figure S1: Fluid dynamics model of intestinal stenosis with the septal formation L: length, D anat: the diameter of the anastmotic site with the septum, Din: the original intestinal diameter. [file ASES-19-e70334-s002.pdf]

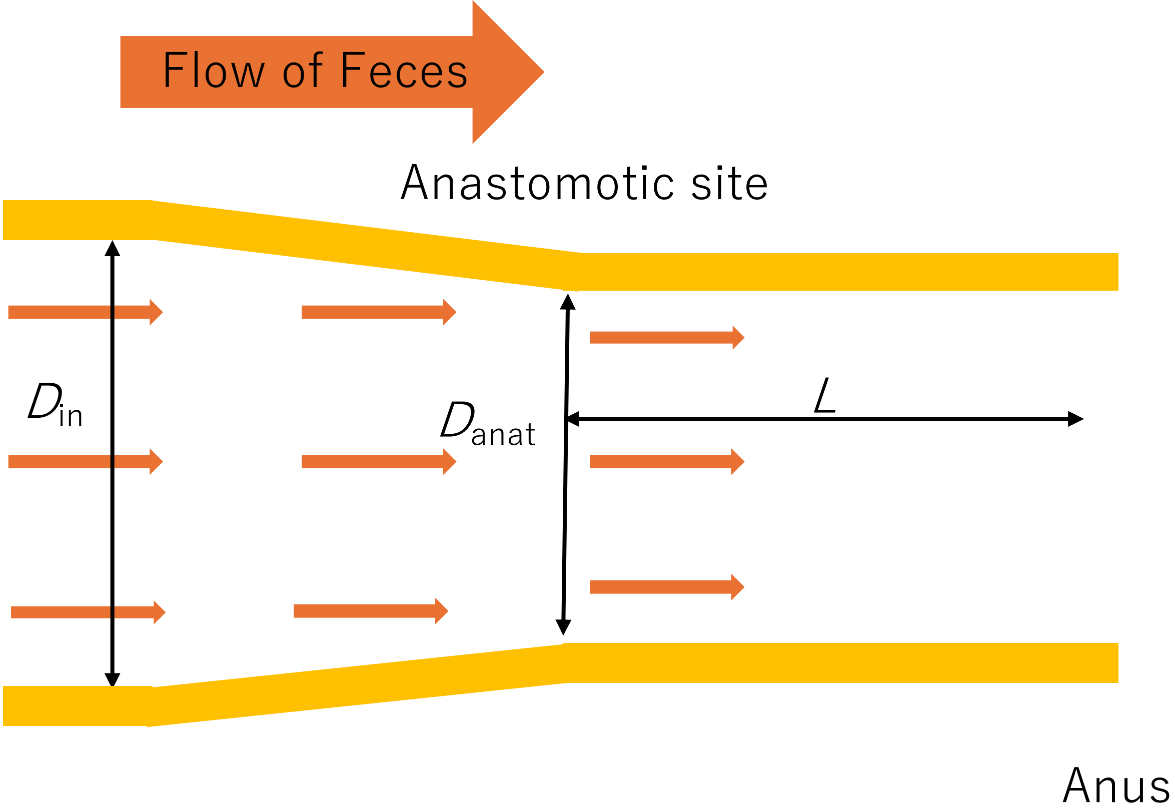

FigureS1 Fluid dynamics model of intestinal stenosis with the septal formation
L: length, D_{anat}: the diameter of the anastmotic site with the septum, D_{in}: the original intestinal diameter
