# Supplementary material for: Endoscopic‐Assisted Division of Septal Formation After Duhamel Procedure for Hirschsprung Disease: Two Case Reports
Source: Asian J Endosc Surg. 2026 Jul 2;19(1):e70334. doi: 10.1111/ases.70334 (PMC13328324; doi:10.1111/ases.70334)
Supplement: Supplementary file 2 — Data S1: Supporting Information. [file ASES-19-e70334-s001.docx]

**Supplemental material**

As a fluid dynamics model of septal formation, we propose a simplified model where a septum reduces intestinal cross-sectional area, while excluding complex factors such as turbulence and alterations in intestinal motility associated with septal formation. In this model, the change in cross-sectional area due to the septum was approximated by the corresponding change in the intestinal radius (Figure S1). Under this assumption, intestinal pressure loss (**∆***P*) can be expressed using a power-law relationship as follows^s1^:

| $\Delta P=K(\frac{4L}{D})(\frac{3n+1}{4n})^{n}(\frac{32V}{\pi D^{3}})^{n}$ | (1) |
| --- | --- |

Here, *K* is the consistency index; $L,$ the length from the anastomosis to the anus, *D,* intestinal diameter; *n,* flow behavior index; and *V,* volumetric flow rate. The pressure loss ratio before and after the septum division serves as an index of the effectiveness of the division (*E*). *E* is defined as:

| $E=\left( \frac{D_{\mathrm{anat}}}{D_{\mathrm{in}}} \right)^{3n+1},$ | (2) |
| --- | --- |

Here, $D_{\mathrm{in}}$ is the original intestinal diameter and $D_{\mathrm{anat}}$ is the intestinal diameter at the anastomotic site stenosed by the septum. *E* is influenced by the flow behavior index (n), which reflects fecal rheology. Typically, n ranges from approximately 0.4 in constipation to 1.0 in diarrhea^s2^. When n approaches 1, as observed in cases of enterocolitis, the pressure loss decreases with the fourth power of the diameter, making septal division more effective under such conditions. Additionally, this formula indicates that when the septal area is small relative to the intestinal dilatation (e.g., $D_{\mathrm{anat}}$/$D_{\mathrm{in}}$ is almost equal to 1), the septal division has minimal effect.

This model did not account for several factors, including the presence of turbulence, flow velocity reduction due to the septum at the center of the anastomotic site, anastomotic diameter, septal width, or the potential decrease in intestinal motility caused by the septum. These factors may play a significant role in pathophysiology. Future studies should incorporate simulations and model development to further investigate these aspects.

**Reference of Supplemental Material**

S1 Metzner ABM, Reed JC. Flow of non-Newtonian fluids – correlation of the laminar, transition, and turbulent-flow regions. *AIChE J.*. 1955;1(4):434–440.

S2 Yang PJ, Lamarca M, Kaminski C, et al. Hydrodynamics of defecation. *Soft Matter*. 2017;13(29):4960.

**Acknowledgements**

We would like to thank Editage ([www.editage.jp](https://www.editage.jp/)) for English language editing.

**Ethical Statement**

This report was conducted in accordance with the ethical guidelines in Japan, which state that institutional review board approval is not necessary for case reports. Written informed consent was obtained from the patients (or their legal guardians) for publication of this report and accompanying images.

**Conflict of interest**

The authors declare no conflict of interest
